# Platinum Nanoparticle Inclusion into a Carbonized Polymer of Intrinsic Microporosity: Electrochemical Characteristics of a Catalyst for Electroless Hydrogen Peroxide Production

**DOI:** 10.3390/nano8070542

**Published:** 2018-07-18

**Authors:** Robert K. Adamik, Naiara Hernández-Ibáñez, Jesus Iniesta, Jennifer K. Edwards, Alexander G. R. Howe, Robert D. Armstrong, Stuart H. Taylor, Alberto Roldan, Yuanyang Rong, Richard Malpass-Evans, Mariolino Carta, Neil B. McKeown, Daping He, Frank Marken

**Affiliations:** 1 Department of Chemistry, University of Bath, Claverton Down, Bath BA2 7AY, UK; adamikr@gmail.com (R.K.A.); y.rong@sussex.ac.uk (Y.R.); 2 Departamento de Química Física e Instituto Universitario de Electroquímica, Universidad de Alicante, Apartado 99, 03080 Alicante, Spain; naiarahbz@gmail.com (N.H.-I.); jesus.iniesta@ua.es (J.I.); 3 Cardiff Catalysis Institute, School of Chemistry, Cardiff University, Main Building, Park Place, Cardiff CF10 3AT, UK; edwardsjk@cardiff.ac.uk (J.K.E.); HoweAG@cardiff.ac.uk (A.G.R.H.); ArmstrongR4@cardiff.ac.uk (R.D.A.); taylorsh@cardiff.ac.uk (S.H.T.); RoldanMartinezA@cardiff.ac.uk (A.R.); 4 East Chem, School of Chemistry, University of Edinburgh, Joseph Black Building, David Brewster Road, Edinburgh, Scotland EH9 3FJ, UK; r.malpassevans@ed.ac.uk (R.M.-E.); neil.mckeown@ed.ac.uk (N.B.M.); 5 Department of Chemistry, Swansea University, College of Science, Grove Building, Singleton Park, Swansea SA2 8PP, UK; mariolino.carta@swansea.ac.uk; 6 Hubei Engineering Research Center of RF-Microwave Technology and Application, School of Science, Wuhan University of Technology, Wuhan 430070, China; dapinghe@hotmail.com

**Keywords:** heterocarbon, microporosity, voltammetry, peroxide, bifunctional catalysis

## Abstract

The one-step vacuum carbonization synthesis of a platinum nano-catalyst embedded in a microporous heterocarbon (Pt@cPIM) is demonstrated. A nitrogen-rich polymer of an intrinsic microporosity (PIM) precursor is impregnated with PtCl_6_^2−^ to give (after vacuum carbonization at 700 °C) a nitrogen-containing heterocarbon with embedded Pt nanoparticles of typically 1–4 nm diameter (with some particles up to 20 nm diameter). The Brunauer-Emmett-Teller (BET) surface area of this hybrid material is 518 m^2^ g^−1^ (with a cumulative pore volume of 1.1 cm^3^ g^−1^) consistent with the surface area of the corresponding platinum-free heterocarbon. In electrochemical experiments, the heterocarbon-embedded nano-platinum is observed as reactive towards hydrogen oxidation, but essentially non-reactive towards bigger molecules during methanol oxidation or during oxygen reduction. Therefore, oxygen reduction under electrochemical conditions is suggested to occur mainly via a 2-electron pathway on the outer carbon shell to give H_2_O_2_. Kinetic selectivity is confirmed in exploratory catalysis experiments in the presence of H_2_ gas (which is oxidized on Pt) and O_2_ gas (which is reduced on the heterocarbon surface) to result in the direct formation of H_2_O_2_.

## 1. Introduction

Nanostructured and microporous composite materials offer new opportunities in catalysis and in electrocatalysis [1]. Electronic effects at the nanoscale affect surface reactivity [2] and can be combined with transport effects that occur in microporous channels and pores in host materials [3]. Recently, it has been shown that hydrophobic pore structures can lead to triphasic reaction conditions (for example in polymers of intrinsic microporosity or PIMs) and that these can also affect electrocatalyst reactivity [4]. In electrocatalysis particularly, carbon-based nano-materials are important, as illustrated by recently developed hetero-carbon materials based on nano-dots [5] and carbon nano-wires or tubes [6]. Access to novel carbon architectures can be achieved, for example, via microporous polymer precursors, which allow functionality such as fluorescence to be retained in the carbon [7] or molecular-scale microporosity to be preserved after gentle carbonization of molecularly rigid polymer precursors [8]. Polymers of intrinsic microporosity provide such molecularly rigid and highly temperature-stable precursor materials that retain porosity and morphology during carbonization [9]. This characteristic can be exploited in the preparation of new microporous composite materials for catalysis.

Polymers of intrinsic microporosity (PIMs) have been developed [10,11] as a new class of molecularly rigid materials with applications in gas capture [12] and separation [13,14,15], electrochemistry [16], and in membrane technologies [17]. The rigid internal channel structure provides control over interactions with guest molecules/particles embedded within the intrinsic micropores with typical pore sizes in the 1–2 nm diameter range [18]. In this range of pore sizes, exclusion effects may occur for some bigger molecules and mobility/diffusion effects may be linked to pore diameter and structure. Higher mobility can be achieved for smaller guest molecules such as H_2_. Based on their special properties, new applications of PIMs have been proposed. Recently, work has been reported focusing in areas such as selective battery membranes [19], fuel cell catalyst corrosion protection [20], enzymeless glucose oxidation [21], switchable membranes [22], triphasic electrocatalysis [23], and for ionic rectification devices [24].

**Figure 1:**
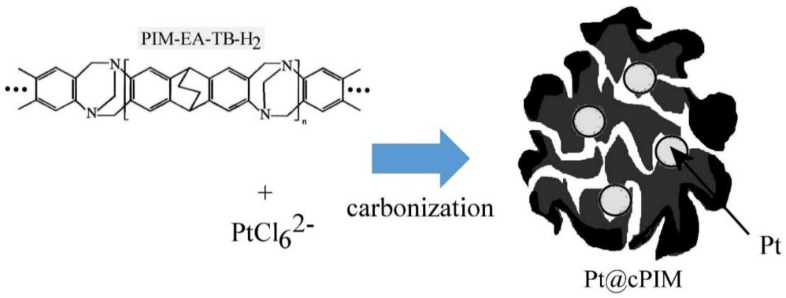
Molecular structure of PIM-EA-TB-H_2_ and schematic depiction of the adsorption of hexachloroplatinic(IV) acid followed by mild vacuum carbonization to give a platinum nano-particle containing microporous heterocarbon.

The molecularly rigid microporous structure of PIMs causes interesting chemical behavior, for example imparting chemical stability under high-temperature thermolysis conditions [8]. Thermolysis of metal-infused PIMs under high-temperature conditions in air has been shown to yield for example nano-structured oxide materials [25]. Under vacuum carbonization conditions the backbone of the PIM material is believed to stay intact (for example for the PIM-EA-TB material [9]) even under conditions of partial carbonization to electrically conducting structures. The heteroatoms (e.g., here nitrogen) can be retained to give heterocarbon materials with high microporosity and with electrical conductivity [8]. Many of the recent studies on applications of PIMs in electrochemistry were based on PIM-EA-TB. The naming of PIM-EA-TB is based on the synthesis route, where an ethanoanthracene “EA” unit is coupled via Tröger Base “TB” coupling [26]. In this report, a similar material, PIM-EA-TB-H_2_, is employed in which two methyl substituents at bridgehead positions have been replaced by hydrogen (see molecular structure in Figure 1).

The vacuum carbonization of PIM materials has been shown to provide novel microporous heterocarbon materials. For similar types of materials, there have already been suggestions for applications in gas separation [27] and in water purification membranes [28]. The electrochemical charging of the microporous carbonized structures has been shown to yield a high double layer capacitance but also pH dependent capacitance phenomena [9]. Two previous reports have indicated that pre-loading PIM-EA-TB with platinum [29] or with palladium [30] can lead to high surface area embedded catalyst materials with active metal nano-particles embedded in the microporous carbon host. A particular benefit arises from the fact that PIMs tend to not contaminate catalyst surfaces during carbonization and therefore additional catalyst activation treatments are not essential. Interest in novel carbon hosts [31] and porous carbon—platinum catalyst composites is strong, for example in fuel cell catalyst development [32,33].

In this report, hexachloroplatinic acid is adsorbed into PIM-EA-TB-H_2_ to give a precursor material that is then vacuum carbonized (at 700 °C) to a novel catalyst hybrid material based on platinum nanoparticles in a microporous carbon. Both the degree of microporosity (controlling gas and charge diffusion) and the electrical conductivity of the material (allowing electron flow from inside to outside of catalyst particles) are shown to be important in the reactivity of the new host-guest catalyst. Electrochemical characterization suggests that only molecular hydrogen (H_2_) diffuses fast enough to reach the embedded catalyst to be oxidized. In exploratory catalysis experiments without external potential control, in the presence of a mixture of hydrogen and oxygen, hydrogen is oxidized to protons within the catalyst micropores, whereas the bulkier oxygen is reduced externally to give hydrogen peroxide in good yield.

## 2. Experimental

### 2.1. Materials and Reagents

All solutions were prepared with deionized water of resistivity not less than 18.2 MΩ cm resistivity taken from a Thermo Scientific water purification unit (ELGA). Methanol, isopropanol, perchloric acid (70%), phosphoric acid (85%), Nafion 117 solution (5 wt %), sodium dihydrogen phosphate (99%) were purchased from Sigma-Aldrich (St. Louis, MO, USA) and used without further purification. Hexachloroplatinic acid (39.89%) obtained from Johnson Matthey Ltd. (London, UK) was used without purification. Laboratory grade (Pureshield) hydrogen gas and oxygen gas cylinders of were obtained from BOC Ltd. Microporous polymer of intrinsic microporosity PIM-EA-TB-H_2_ was synthesized following the literature recipe [26,34].

### 2.2. Instrumentation

Carbonization was performed in a quartz tube linked to a vacuum pump (oil) in a TSH12 tubular furnace (Elite Thermal System Ltd., Market Harborough, UK). Carbonized samples were characterized by transmission electron microscopy (TEM) on a Joel JEM-2100 Plus TEM system with PED (Preccesion Electron Diffraction). Porosity analysis was performed with samples of 20 mg, which were degassed for six h at 120 °C prior to analysis. Analysis was carried with N_2_ at 77 K on a Micrometrics 3Flex with P_0_ measured continuously. Free space analysis with He was measured post-analysis. Recorded 80-point adsorption isotherm was analyzed with density functional theory (DFT) method (N_2_ at 77 K, cylindrical pore shape, NLDFT equilibrium model). Carbon monoxide (CO) chemisorption measurements were performed using a Quantachrome ChemBET TPR/TPD chemisorption analyzer (Quantachrome Instruments, Boynton Beach, FL, USA) fitted with a TCD and 70 μL injection loop. Prior to chemisorption of CO, samples were pretreated (200 °C, 2 h) in a flow of H_2_ (30 mL min^−1^ STP). Platinum dispersion was calculated from total CO uptake assuming a 1:1 stoichiometry of adsorbed CO: surface Pt atoms [35]. Elemental analysis (obtained by Butterworth Laboratories Ltd., London, UK) suggests for Pt@cPIM Pt 26% C 55.07% H 1.10% N 4.75%, which compares to the previously reported cPIM C 83.19% H 2.46% N 5.67% [9].

Electrochemical characterization was performed with either an Ivium *Compactstat* potentiostat or a μAutolab type III potentiostat-galvanostat in a three-electrode configuration and in a conventional glass cell. A platinum wire was used as the counter electrode, KCl-saturated calomel (saturated calomel electrode, SCE, Radiometer) was used as the reference electrode, and a catalyst modified 3 mm diameter glassy carbon disc electrode (GC, BASi) was used as the working electrode.

### 2.3. Catalyst and Electrode Preparation

Powdered PIM-EA-TB-H_2_ (21 mg) was first impregnated with platinum by immersion into an excess (10 mL) of 10 mM hexachloroplatinic acid solution in methanol. The sample was allowed to soak in the solution for 24 h. The sample was then centrifuged (8000 rpm, 15 min), decanted and washed with 10 mL of water. The washing step was repeated and then the yellow-colored polymer sample was left to dry at ambient temperature and pressure for 24 h. Carbonization was performed at 700 °C for 3 h in a custom-made evacuated quartz tube heated in a tubular furnace (in an oil pump vacuum of approximately 4 mbar). After cooling down an air-stable black powder is obtained.

Catalyst ink was prepared with 2 mg of the carbonized powder material, which was homogenized by grinding (manually with mortar and pestle) together with 100 µL of Nafion 117 solution (5 wt %), then dispersed in 1 mL of isopropanol and sonicated for 15 min until fully homogeneous. A small volume (between 5–15 µL, as required) was drop-casted onto the 3 mm glassy carbon disk electrode and allowed to ambiently dry before electrochemical analysis.

### 2.4. Catalyst Testing

Catalysts were tested for their direct hydrogen peroxide synthesis activity using a stainless-steel autoclave (Parr). The 100 mL autoclave was fitted with a Teflon liner—nominal volume 66 mL. In a typical synthesis test, the autoclave was charged with catalyst (0.01 g), water (2.9 g) and methanol (5.6 g), then purged with 5% H_2_/CO_2_ three times before being filled with 5% H_2_/CO_2_ (29 bar) then 25% O_2_/CO_2_ (11 bar). The autoclave was chilled to 2 °C before stirring at 1200 rpm for 30 min. After completion of the reaction, H_2_O_2_ formation is detected quantitatively.

## 3. Results and Discussion

### 3.1. Properties of Pt@cPIM I.: Structure and Porosity

**Figure 2:**
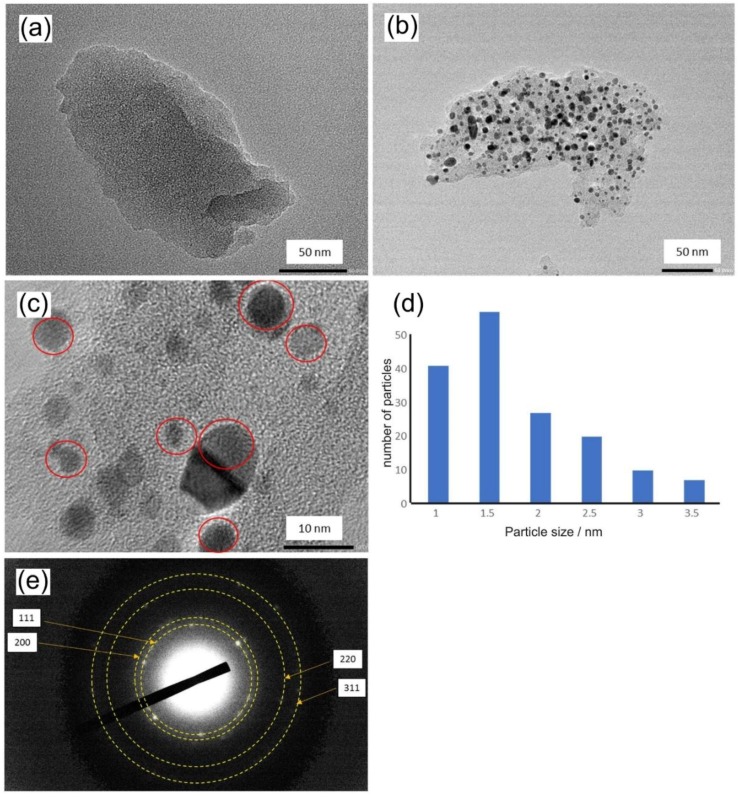
Transmission electron microscopy images for (**a**) carbonized hexachloroplatinate loaded polymer of intrinsic microporosity (PIM-EA-TB-H_2_), (**b**) carbonized PIM-EA-TB-H_2_ with embedded nano-platinum, (**c**) high magnification micrograph showing platinum crystal lattice planes, (**d**) particle size distribution analysis, and (**e**) electron diffractogram with characteristic *hkl* reflections for platinum face-centered cubic phase.

Successful impregnation of polymer by hexachloroplatinate precursor and formation of platinum nanoparticles during carbonization is confirmed by comparison of transmission electron microscopy (TEM (Transmission Electron Microscopy)) images of carbonized polymer PIM-EA-TB-H_2_ without platinum (see Figure 2a) and the new Pt@cPIM catalyst material (see Figure 2b). Particle size distribution data for platinum nanoparticles (see Figure 2d) show that the majority of particles falls into a 1–4 nm diameter size range with some bigger particles up to 20 nm. High magnification images reveal high crystallinity of platinum nanoparticles with clear presence of crystal lattice planes (see Figure 2c) consistent with platinum metal nanoparticles.

Electron diffraction data (Figure 2e) confirm the presence of crystalline platinum nano-particles. Diffraction rings typical for platinum face-centered cubic phase are observed. The thermolysis of PIM-EA-TB-H_2_ without metal inclusions was reported recently [9] to give a similar material, although without platinum inclusion. Thermogravimetric data suggested that even at temperatures up to 700 °C the weight loss was limited to about 26%. Both Raman spectra and electrochemical properties suggested that an electrically conducting sp^2^-carbon-rich material was formed. The nitrogen gas adsorption isotherm was reported indicative of a loss of Brunauer-Emmett-Teller (BET) surface area from 846 m^2^ g^−1^ for pure PIM-EA-TB-H_2_ to 425 m^2^ g^−1^ for the carbonized PIM-EA-TB-H_2_ (cPIM). However, the cumulative pore volume for the range from 1 nm to 5 nm remained similar before and after carbonization.

**Figure 3:**
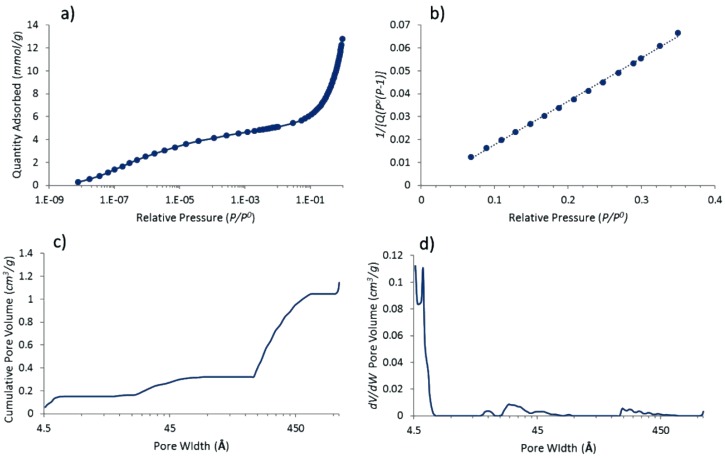
BET (77 K) nitrogen adsorption isotherm results for platinum nano-catalyst embedded in a microporous heterocarbon (Pt@cPIM): (**a**) N_2_ adsorption isotherm; (**b**) BET analysis plot; (**c**) cumulative pore volume; (**d**) pore width distribution (NLDFT (Non-Localised Density Functional Theory)) equilibrium for cylindrical pore model).

Gas adsorption and porosity characterization were performed for Pt@cPIM based on the nitrogen adsorption isotherm at 77 K (see Figure 3a). Data indicate at least partial retention of original microporous polymer structure in the carbonized Pt@cPIM catalyst material. This observation agrees with previous reports regarding vacuum carbonization of PIM-EA-TB at 500 °C [8]. The calculated BET (Brunauer-Emmet-Teller) surface area of 518 m^2^ g^−1^ (Figure 3b) is lower than the 846 m^2^ g^−1^ reported for the pure PIM-EA-TB-H_2_ polymer and relatively close to the 425 m^2^ g^−1^ reported recently for the carbonized polymer without platinum [9]. The data imply that vacuum carbonization did not lead to a significant loss of small micropores. Impregnation of the polymer with the platinum precursor led to the formation of platinum nanoparticles without strongly affecting the properties of the microporous host carbon. Data from elemental analysis for Pt@cPIM (Pt 26% C 55.07% H 1.10% N 4.75%) confirm that nitrogen has been retained to an extent similar to that for the previously reported cPIM C 83.19% H 2.46% N 5.67% [9]. These backbone-incorporated nitrogen functionalities have been shown to be associated with protonation and pH-dependent variation in capacitance when immersed in aqueous solution [9].

### 3.2. Properties of Pt@cPIM II.: Electrochemical Characterization

**Figure 4:**
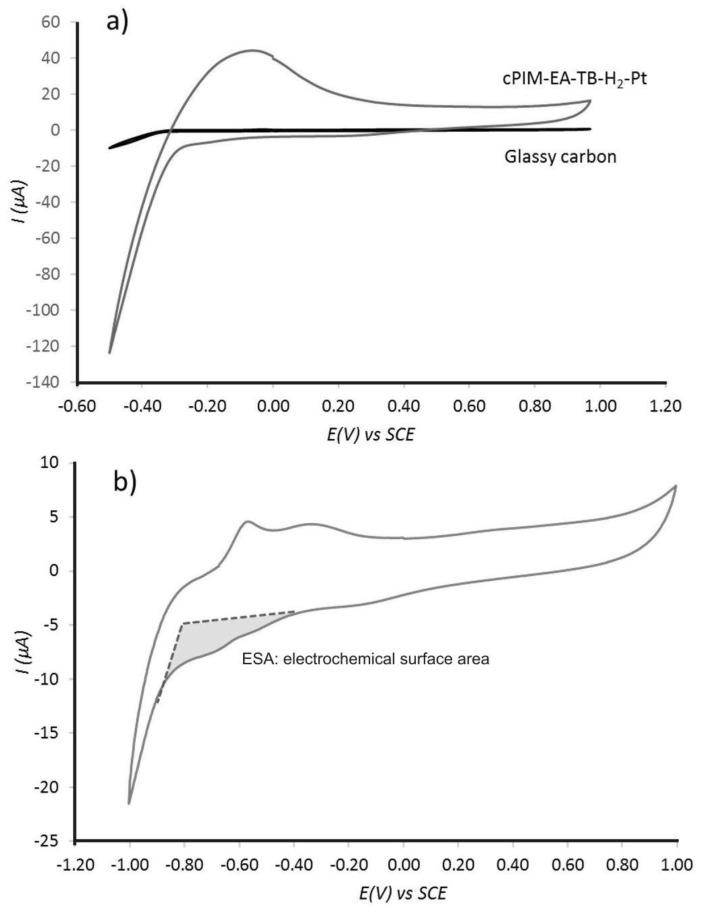
Cyclic voltammograms (3rd cycle, Ar saturated, scan rate 10 mV s^−1^) at Pt@cPIM immobilized onto a 3 mm diameter glassy carbon electrode immersed into (**a**) 0.1 M HClO_4_ solution and (**b**) 10 mM phosphate buffer pH 7 solution with highlighted hydrogen electrochemical underpotential adsorption region (grey).

Electrochemical characterization was performed with 3 mm diameter glassy carbon working electrode, coated with a volume (typically 5–15 μL) of ink (see experimental) applied by drop-casting. The electrochemical activity of the nano-platinum embedded in the carbonized polymer is evident when Pt@cPIM voltammograms are compared with a voltammogram recorded in aqueous 0.1 M HClO_4_ for the bare glassy carbon electrode (see Figure 4a). A substantial capacitive current component can be attributed to the microporous carbon and the distinct proton reduction and hydrogen oxidation response at −0.3 V vs. SCE clearly demonstrates catalytic activity.

Identification of characteristic hydrogen underpotential adsorption peaks [36] was not possible in acidic aqueous solution due to a high charging current generated by the capacitive nature of the cPIM-electrolyte interface. The presence of excess protons is believed to increase the double layer capacitance [9]. Therefore, the determination of the electrochemically active surface area for platinum (ESA) from the charge required to adsorb a hydrogen monolayer was estimated for an electrode immersed in 10 mM phosphate buffer solution at pH 7, where the hydrogen underpotential adsorption was more defined (Figure 4b) and the capacitive charging current was lower. Estimation of electrochemically active surface area with $ESA=\frac{Q_{H}}{210\;\mu C\;{\mathrm{cm}}^{-2}}$ (where *Q_H_* is the charge required to remove hydrogen monolayer [37]) gave a specific ESA of 5.6 m^2^ g^−1^_,_ which has to be regarded as a rough estimate. Additional experiments were performed employing carbon monoxide adsorption/desorption measurement (see experimental). In this case, a value of the platinum surface area of 2.15 m^2^ g^−1^ was obtained in reasonable agreement with the electrochemical surface area estimate.

**Figure 5:**
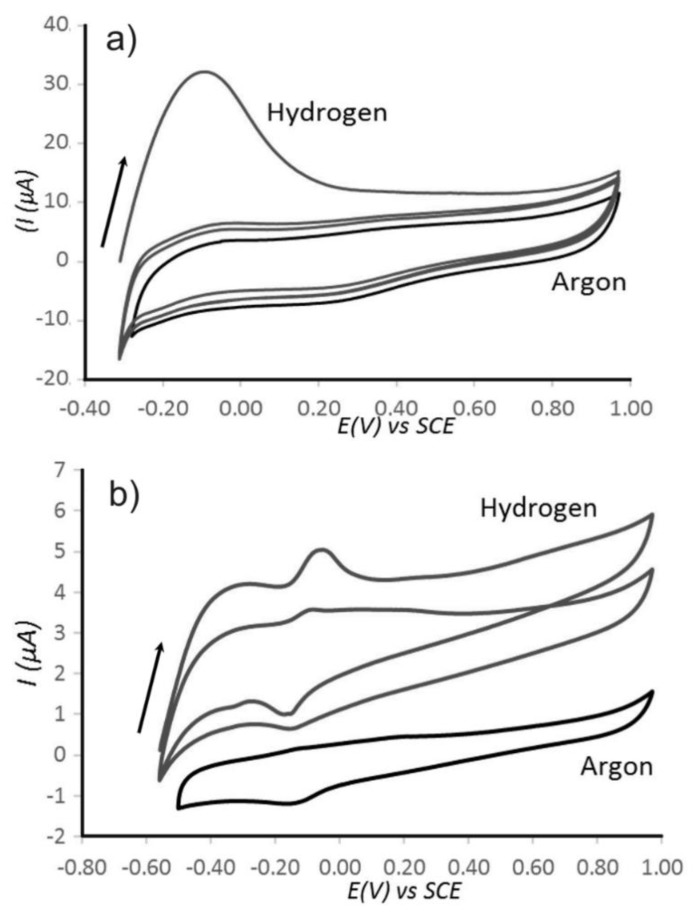
Cyclic voltammograms (scan rate 10 mV s^−1^) at Pt@cPIM immobilized onto a 3 mm diameter glassy carbon electrode immersed into (**a**) aqueous 0.1 M HClO_4_ and (**b**) aqueous 10 mM PBS pH 7 solution purged with Ar or H_2_.

***Hydrogen Oxidation Reaction (HOR).*** Hydrogen evolution [38,39,40] and hydrogen oxidation represent an exceptionally thoroughly studied class of electrocatalytic processes [41]. New types of catalysts are still being developed [42]. The electrocatalytic hydrogen oxidation was performed here with use of a catalyst ink drop-casted onto the glassy carbon electrode, which is then immersed into a hydrogen saturated 0.1 M perchloric acid solution (Figure 5a) and into 10 mM phosphate buffered saline (PBS (Phosphate Buffered Saline), pH 7) solution (Figure 5b). The process is assumed to occur via two-electron oxidation (Equation (1)).

(1) $$H_{2}\to 2H^{+}+2e^{-}$$

Cyclic voltammograms for hydrogen and argon saturated solutions were compared. Hydrogen oxidation is clearly observed at potential positive of approximately −0.3 V vs. SCE in aqueous 0.1 M HClO_4_ (see Figure 5a) and at potentials positive of −0.5 V vs. SCE in phosphate buffer at pH 7 (note the shift in the onset potential comparing Figures 4b and 5b probably due to some acidification within the pores of the catalyst particles). In addition to the onset potentials for hydrogen evolution, double-peak features are observed especially in the presence of phosphate buffer (Figure 5b, compare to Figure 4b). These chemically reversible peaks in voltammograms occur positive of the bulk hydrogen region and are consistent with the under-potential deposition (UPD) hydrogen region on platinum surfaces [43]. Multi-cycle voltammograms in 0.1 M HClO_4_ show an initial peak followed by constant hydrogen oxidation with about 3 to 4 A current (Figure 5a). In phosphate buffer solution (Figure 5b) the hydrogen underpotential adsorption peaks are clearly observed and efficient hydrogen oxidation is reflected in a relatively high hydrogen desorption current peak at approximately −0.1 V vs. SCE (Saturated Calomel Electrode). The hydrogen oxidation current is again approximately 3 to 4 A (compare Figure 5a,b) and therefore probably limited by the rate of hydrogen diffusion into the Pt@cPIM particles at the electrode surface.

From the capacitive current background response and the known amount of deposit, the specific capacitance of the Pt@cPIM material can be estimated (employing capacitance = *I*_capacitance_/scan rate) as 5 Fg^−1^ in neutral phosphate buffer solution and 27 Fg^−1^ in 0.1 M perchloric acid. These values compare well with the recently reported value of 40 Fg^−1^ for cPIM in 0.1 M perchloric acid [9].

***Methanol Oxidation Reaction (MOR).*** The methanol oxidation reaction is investigated here as a model reaction that is important in many fuel cell applications. Although various types of catalysts systems have been studied [44,45], platinum has been shown to be an effective catalyst for methanol fuel cells [46]. Methanol oxidation is a complex catalytic reaction with multiple reaction steps and an overall reaction shown in Equation (2).

(2) $${\mathrm{CH}}_{3}\mathrm{OH}+H_{2}O\to {\mathrm{CO}}_{2}+6H^{+}+6e^{-}$$

**Figure 6:**
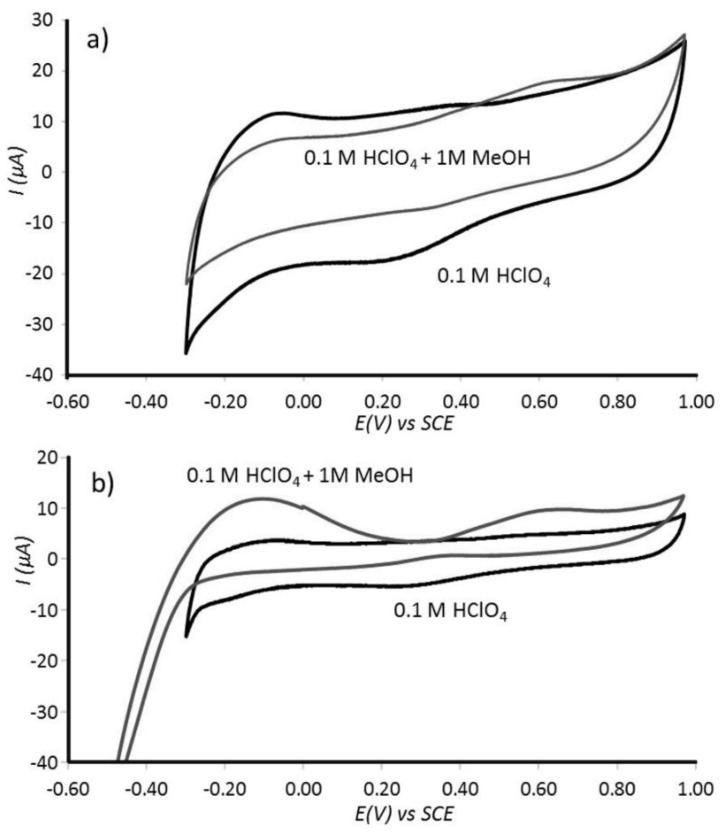
Cyclic voltammograms (3rd cycle) at Pt@cPIM immobilized onto a 3 mm diameter glassy carbon electrode immersed into Ar saturated 0.1 M HClO_4_ without/with 1.0 M methanol recorded at a scan rate of (**a**) 100 mV s^−1^ and (**b**) 10 mV s^−1^.

Voltammograms for methanol oxidation recorded at 0.1 V s^−1^ scan rate in aqueous 0.1 M HClO_4_ are presented in Figure 6. Only a very minor methanol oxidation current peak is apparent at approximately 0.7 V vs. SCE in the forward cycle. At a lower scan rate the methanol oxidation can be identified more clearly (Figure 6b), but catalytic currents are obviously very low even for 1 M methanol. This result suggests a restricted access of methanol molecules to the nano-platinum active sites. This, therefore, suggests high selectivity towards the smaller hydrogen molecules.

***Oxygen Reduction Reaction (ORR).*** Oxygen reduction is a crucial process in many areas of energy technology. Oxygen reduction catalysts have been developed based on noble metals, transition metals [47,48], oxides/hydroxides [49,50], and other types of catalytic materials. The ability to separate oxygen reduction from hydrogen oxidation is fundamental in fuel cells electrochemistry. Therefore, here the selectivity of platinum nanoparticles embedded in carbonized microporous polymer (Pt@cPIM) towards oxygen electro-reduction was investigated and cyclic voltammetry experiments were performed. There are two distinct reaction products possible linked to the 4-electron reduction (see Equation (3), predominant on platinum) and linked to the 2-electron reduction (see Equation (4), predominant on carbon).

(3) $$O_{2}+4H^{+}+4e^{-}\to 2H_{2}O$$

(4) $$O_{2}+2H^{+}+2e^{-}\to H_{2}O_{2}$$

**Figure 7:**
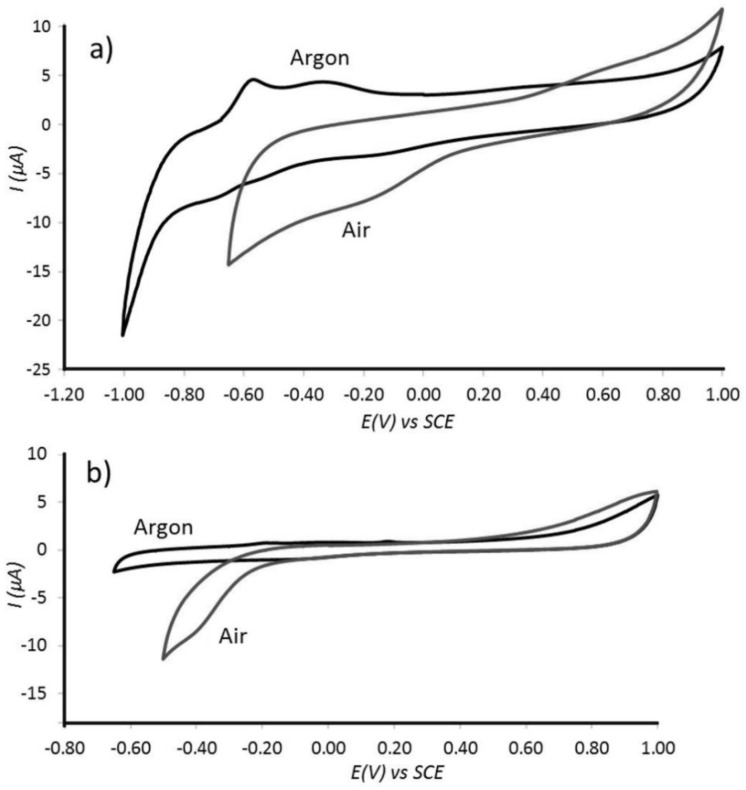
Cyclic voltammogram (3rd cycle, scan rate (**a**) 10 mV s^−1^ and (**b**) 5 mV s^−1^) for oxygen reduction at Pt@cPIM immobilized onto a 3 mm diameter glassy carbon electrode immersed into 10 mM phosphate buffer solution pH 7 purged with either Ar or air.

The catalyst ink material was drop-casted onto a 3 mm diameter glassy carbon disk electrode and immersed in oxygen saturated 10 mM phosphate buffer solution at pH 7 (see Figure 7a,b). The oxygen reduction peak can be identified at approximately −0.4 V vs. SCE and its magnitude suggests effective reduction. At a slightly higher potential scan rate (Figure 7a) a smaller peak at −0.2 V vs. SCE is observed, but probably not associated with oxygen reduction. The reduction of oxygen at −0.4 V vs. SCE occurs at very negative potential more typical for that of the 2-electron reduction on carbon materials [4]. This result is striking in that there is very little catalytic ability of the platinum towards oxygen reduction under these conditions. High selectivity towards hydrogen oxidation is again achieved and oxygen reduction is suppressed probably due to pore size effects. Further effects could be associated with the pH inside of the Pt@cPIM carbon particles, with hydrogen oxidation causing acidic conditions and protonation of nitrogen groups in Pt@cPIM in contrast to oxygen reduction causing alkaline conditions and a change in the pore properties. The reduction of oxygen on carbon shell (and on the glassy carbon support) is likely to produce hydrogen peroxide (H_2_O_2_) instead of water.

### 3.3. Properties of Pt@cPIM III.: Catalysis and Hydrogen Peroxide Formation

**Figure 8:**
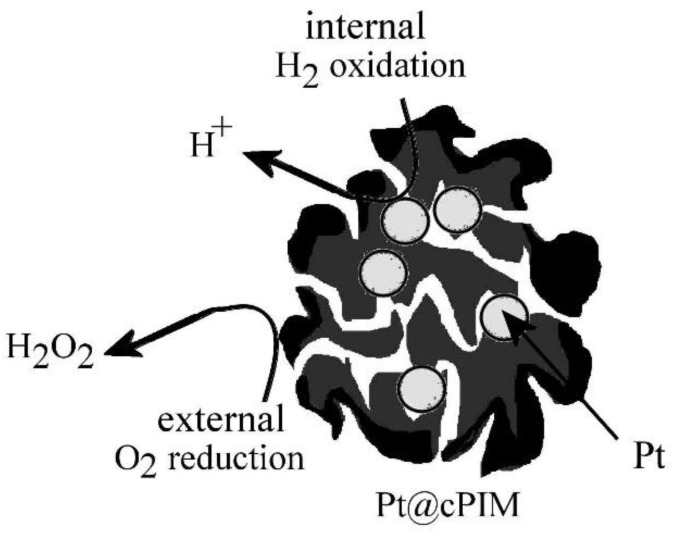
Schematic depiction of the catalytic process involving hydrogen and oxygen to give hydrogen peroxide.

Although direct electrochemical methods for hydrogen peroxide production are possible (e.g., based on driven fuel cells [51]), here the reactivity of the Pt@cPIM material is investigated for the direct non-electrochemical catalytic conversion of hydrogen and oxygen to H_2_O_2_. This homogeneous-catalytic conversion of hydrogen and oxygen directly to hydrogen peroxide is of considerable interest and novel alloy catalyst systems have been reported previously [52]. Figure 8 depicts the schematic reaction as a process, in which molecular hydrogen is able to penetrate into the catalyst particles and react at the surface of the platinum particles. As a result, electrons are suggested to be generated internally and conducted to the surface of the Pt@cPIM catalyst particle. The oxygen reduction is believed to then occur only at the surface to give mainly H_2_O_2_. When evaluated for the direct synthesis reaction under standard catalytic reaction conditions, an activity of 14 mol_H2O2_ kg_cat_^−1^ h^−1^ was achieved. This result is considerably higher than that observed for a 5 wt % Pt/TiO_2_ prepared by impregnation [53] and is comparable to the activity of some mono-metallic Pd catalysts, which tend to be most active for the direct synthesis reaction. Further work will be required to better compare and improve the performance of the novel Pt@cPIM catalyst materials.

(5) $$O_{2}+H_{2}\to H_{2}O_{2}$$

## 4. Conclusions

A novel composite catalyst has been prepared based on platinum nanoparticles embedded into a microporous heterocarbon. A one-step synthesis is employed based on the carbonization of a hexachloroplatinate loaded polymer of intrinsic microporosity (PIM-EA-TB-H_2_), which at 700 °C give the microporous catalyst with bi-functional reactivity and molecular size selectivity. The special nature of PIM materials with (A) molecular rigidity contributing to high temperature stability and with (B) molecular backbone and porosity being maintained provide a way of preparing embedded platinum catalysts in nano-size and without significant surface contamination. The catalysts are ready to use without further activation treatments. In electrochemical measurements it was revealed that hydrogen oxidation occurs as expected for a platinum catalyst, but oxygen reduction and methanol oxidation are suppressed due to the microporosity of the heterocarbon environment. Protonation of the nitrogen functionalities in the porous carbon during hydrogen oxidation may also contribute to the observed selectivity.

The testing of catalytic activity (in particular for H_2_ + O_2_ reactivity) is preliminary in nature and optimization of the catalyst performance based on Pt loading, carbonization temperature, particle shape and size etc. is likely to be possible. Instead of a powder, the catalyst could be applied as a thin film on an inert substrate to be re-used or as part of a continuous reactor system. Further work will be needed to investigate the catalyst after use in catalysis (to confirm the nature of the catalyst) and to develop catalyst re-cycling procedures.

Generally, more study will be needed to further develop the field of PIM precursors for heterocarbon and catalyst materials. The intrinsic microporosity and thermal stability of PIMs allows highly interesting microporous hybrid materials to be obtained in a single step without activation and with embedded catalyst particles in an active state (the surface not blocked by the carbonization process). Catalysts could be developed for a wider range of applications.

## References

[1] Lavacchi A., Miller H., Vizza F. (2013). Nanotechnology in Electrocatalysis for Energy.

[2] Guo S.J., Zhang S., Sun S.H. (2013). Tuning nanoparticle catalysis for the oxygen reduction reaction. Angew. Chem. Int. Ed..

[3] Nguyen T.D., Dinh C.T., Do T.O. (2015). Tailoring the assembly, interfaces, and porosity of nanostructures toward enhanced catalytic activity. Chem. Commun..

[4] Madrid E., Lowe J.P., Msayib K.J., McKeown N.B., Song Q., Attard G.A., Düren T., Marken F. (2018). Triphasic nature of polymers of intrinsic microporosity induces storage and catalysis effects in hydrogen and oxygen reactivity atelectrode surfaces. ChemElectroChem.

[5] Gao J., Zhu M.M., Huang H., Liu Y., Kang Z.H. (2017). Advances, challenges and promises of carbon dots. Inorg. Chem. Front..

[6] Dumitrescu I., Unwin P.R., Macpherson J.V. (2009). Electrochemistry at carbon nanotubes: Perspective and issues. Chem. Commun..

[7] Lawrence K., Xia F.J., Arrowsmith R.L., Ge H.B., Nelson G.W., Foord J.S., Felipe-Sotelo M., Evans N.D.M., Mitchels J.M., Flower S.E. (2014). Hydrothermal conversion of one-photon-fluorescent poly(4-vinylpyridine) into two-photon-fluorescent carbon nanodots. Langmuir.

[8] Rong Y.Y., He D.P., Sanchez-Fernandez A., Evans C., Edler K.J., Malpass-Evans R., Carta M., McKeown N.B., Clarke T.J., Taylor S.H. (2015). Intrinsically microporous polymer retains porosity in vacuum thermolysis to electroactive heterocarbon. Langmuir.

[9] Hernandez N., Iniesta J., Leguey V.M., Armstrong R., Taylor S.H., Madrid E., Rong Y.Y., Castaing R., Malpass-Evans R., Carta M. (2017). Carbonization of polymers of intrinsic microporosity to microporous heterocarbon: Capacitive pH measurements. Appl. Mater. Today.

[10] Qiu S.L., Ben T. (2016). Polymers of instrinsic microporosity. Porous Polymers: Design, Synthesis and Applications.

[11] McKeown N.B., Budd P.M. (2006). Polymers of intrinsic microporosity (PIMs): Organic materials for membrane separations, heterogeneous catalysis and hydrogen storage. Chem. Soc. Rev..

[12] Ullah R., Atilhan M., Anaya B., Al-Muhtaseb S., Aparicio S., Patel H., Thirion D., Yavuz C.T. (2016). Investigation of ester-and amide-linker-based porous organic polymers for carbon dioxide capture and separation at wide temperatures and pressures. ACS Appl. Mater. Interfaces.

[13] Ramimoghadam D., Gray E.M., Webb C.J. (2016). Review of polymers of intrinsic microporosity for hydrogen storage applications. Int. J. Hydrogen Energy.

[14] Polak-Krasna K., Dawson R., Holyfield L.T., Bowen C.R., Burrows A.D., Mays T.J. (2017). Mechanical characterization of polymer of intrinsic microporosity PIM-1 for hydrogen storage applications. J. Mater. Sci..

[15] Ghanem B.S., McKeown N.B., Budd P.M., Al-Harbi N.M., Fritsch D., Heinrich K., Starannikova L., Tokarev A., Yampolskii Y. (2009). Synthesis, characterization, and gas permeation properties of a novel group of polymers with intrinsic microporosity: PIM-polyimides. Macromolecules.

[16] Rong Y.Y., Malpass-Evans R., Carta M., McKeown N.B., Attard G.A., Marken F. (2014). High density heterogenization of molecular electrocatalysts in a rigid intrinsically microporous polymer. Electrochem. Commun..

[17] Song Q.L., Cao S., Pritchard R.H., Ghalei B., Al-Muhtaseb S.A., Terentjev E.M., Cheetham A.K., Sivaniah E. (2014). Controlled thermal oxidative crosslinking of polymers of intrinsic microporosity towards tunable molecular sieve membranes. Nat. Commun..

[18] McKeown N.B., Budd P.M. (2010). Exploitation of intrinsic microporosity in polymer-based materials. Macromolecules.

[19] Thomas A., Kuhn P., Weber J., Titirici M.M., Antonietti M. (2009). Porous polymers: Enabling solutions for energy applications. Macromol. Rapid Commun..

[20] He D.P., Rong Y.Y., Kou Z.K., Mu S.C., Peng T., Malpass-Evans R., Carta M., McKeown N.B., Marken F. (2015). Intrinsically microporous polymer slows down fuel cell catalyst corrosion. Electrochem. Commun..

[21] Rong Y.Y., Malpass-Evans R., Carta M., McKeown N.B., Attard G.A., Marken F. (2014). Intrinsically porous polymer protects catalytic gold particles for enzymeless glucose oxidation. Electroanalysis.

[22] Madrid E., Rong Y.Y., Carta M., McKeown N.B., Malpass-Evans R., Attard G.A., Clarke T.J., Taylor S.H., Long Y.T., Marken F. (2014). Metastable ionic diodes derived from an amine-based polymer of intrinsic microporosity. Angew. Chem. Int. Ed..

[23] Leong S.X., Carta M., Malpass-Evans R., McKeown N.B., Madrid E., Marken F. (2018). One-step preparation of microporous Pd@cPIM composite catalyst film for triphasic electrocatalysis. Electrochem. Commun..

[24] Rong Y.Y., Song Q.L., Mathwig K., Madrid E., He D.P., Niemann R.G., Cameron P.J., Dale S.E.C., Bending S., Carta M. (2016). Rectifier pH-induced reversal of ionic diode polarity in 300 nm thin membranes based on a polymer of intrinsic microporosity. Electrochem. Commun..

[25] Al-Kutubi H., Rassaei L., Olthuis W., Nelson G.W., Foord J.S., Holdway P., Carta M., Malpass-Evans R., McKeown N.B., Tsang S.C. (2015). Polymers of intrinsic microporosity as high temperature templates for the formation of nanofibrous oxides. RSC Adv..

[26] Carta M., Malpass-Evans R., Croad M., Rogan Y., Jansen J.C., Bernardo P., Bazzarelli F., McKeown N.B. (2013). An efficient polymer molecular sieve for membrane gas separations. Science.

[27] Salinas O., Ma X.H., Litwiller E., Pinnau I. (2016). High-performance carbon molecular sieve membranes for ethylene/ethane separation derived from an intrinsically microporous polyimide. J. Membr. Sci..

[28] Kim H.J., Kim D.G., Lee K., Baek Y., Yoo Y., Kim Y.S., Kim B.G., Lee J.C. (2016). A carbonaceous membrane based on a polymer of intrinsic microporosity (PIM-1) for water treatment. Sci. Rep..

[29] Rong Y.Y., He D.P., Malpass-Evans R., Carta M., McKeown N.B., Gromboni M.F., Mascaro L.H., Nelson G.W., Foord J.S., Holdway P. (2017). High-utilization nanoplatinum catalyst (Pt@cPIM) obtained via vacuum carbonization in a molecularly rigid polymer of intrinsic microporosity. Electrocatalysis.

[30] Xia F.J., Pan M., Mu S.C., Malpass-Evans R., Carta M., McKeown N.B., Attard G.A., Brew A., Morgan D.J., Marken F. (2014). Polymers of intrinsic microporosity in electrocatalysis: Novel pore rigidity effects and lamella palladium growth. Electrochim. Acta.

[31] You P.Y., Kamarudin S.K. (2017). Recent progress of carbonaceous materials in fuel cell applications: An overview. Chem. Eng. J..

[32] Afraz A., Rafati A.A., Hajian A., Khoshnood M. (2015). Electrodeposition of Pt nanoparticles on new porous graphitic carbon nanostructures prepared from biomass for fuel cell and methanol sensing applications. Electrocatalysis.

[33] Kakaei K. (2012). Electrochemical characteristics and performance of platinum nanoparticles supported by Vulcan/polyaniline for oxygen reduction in PEMFC. Fuel Cells.

[34] Bernardo P., Scorzafave V., Clarizia G., Tocci E., Jansen J.C., Borgogno A., Malpass-Evans R., McKeown N.B., Carta M., Tasselli F. (2018). Thin film composite membranes based on a polymer of intrinsic microporosity derived from Tröger’s base: A combined experimental and computational investigation of the role of residual casting solvent. J. Membr. Sci.

[35] Lowell S., Shields J.E., Thomas M.A., Thommes M. (2004). Characterization of Porous Solids and Powders: Surface Area, Pore Size and Density.

[36] Weber J., Wain A.J., Attard G.A., Marken F. (2017). Electrothermal Annealing of Catalytic Platinum Microwire Electrodes: Towards Membrane-Free pH 7 Glucose Micro-Fuel Cells. Electroanalysis.

[37] Biegler T., Rand D.A.J., Woods R. (1971). Limiting oxygen coverage on platinized platinum; relevance to determination of real platinum area by hydrogen adsorption. J. Electroanal. Chem..

[38] Mtukula A.C., Bo X.J., Guo L.P. (2017). Highly active non-precious metal electrocatalyst for the hydrogen evolution reaction based on nitrogen-doped graphene supported MoO_2_/WN/Mo_2_N. J. Alloys Compd..

[39] Zhou W.J., Jia J., Lu J., Yang L.J., Hou D.M., Li G.Q., Chen S.W. (2016). Recent developments of carbon-based electrocatalysts for hydrogen evolution reaction. Nano Energy.

[40] Yu Z.T., Ye J.B., Chen W.X., Xu S.R. (2017). Fabrication of MoS_2_/reduced graphene oxide hybrid as an earth-abundant hydrogen evolution electrocatalyst. Mater. Lett..

[41] Sheng W.C., Gasteiger H.A., Shao-Horn Y. (2010). Hydrogen oxidation and evolution reaction kinetics on platinum: Acid vs. alkaline electrolytes. J. Electrochem. Soc..

[42] Wang D.Z., Shen Y.L., Zhang X.Y., Wu Z.Z. (2017). Enhanced hydrogen evolution from the MoP/C hybrid by the modification of Ketjen black. J. Mater. Sci..

[43] Hotchen C.E., Attard G.A., Bull S.D., Marken F. (2014). One-step electroless growth of nano-fibrous platinum catalyst from “paint-on” PtCl62-solution in poly-(ethylene-glycol). Electrochim. Acta.

[44] Candelaria S.L., Bedford N.M., Woehl T.J., Rentz N.S., Showalter A.R., Pylypenko S., Bunker B.A., Lee S., Reinhart B., Ren Y., Ertem S.P. (2017). Multi-component Fe-Ni hydroxide nanocatalyst for oxygen evolution and methanol oxidation reactions under alkaline conditions. ACS Catal..

[45] Yang C.Z., van der Laak N.K., Chan K.Y., Zhang X. (2012). Microwave-assisted microemulsion synthesis of carbon supported Pt-WO3 nanoparticles as an electrocatalyst for methanol oxidation. Electrochim. Acta.

[46] He D.P., Rong Y.Y., Carta M., Malpass-Evans R., McKeown N.B., Marken F. (2016). Fuel cell anode catalyst performance can be stabilized with a molecularly rigid film of polymers of intrinsic microporosity (PIM). RSC Adv..

[47] Gupta S., Qiao L., Zhao S., Xu H., Lin Y., Devaguptapu S.V., Wang X.L., Swihart M.T., Wu G. (2016). Highly active and stable graphene tubes decorated with FeCoNi alloy nanoparticles via a template-free graphitization for bifunctional oxygen reduction and evolution. Adv. Energy Mater..

[48] Wu H.J., Guo C.Z., Li J.Q., Ma Z.L., Feng Q.Y., Chen C.G. (2016). A graphene-based electrocatalyst co-doped with nitrogen and cobalt for oxygen reduction reaction. Int. J. Hydrogen Energy.

[49] Song W.Q., Ren Z., Chen S.Y., Meng Y.T., Biswas S., Nandi P., Elsen H.A., Gao P.X., Suib S.L. (2016). Ni- and Mn-promoted mesoporous Co_3_O_4_: A stable bifunctional catalyst with surface-structure-dependent activity for oxygen reduction reaction and oxygen evolution reaction. ACS Appl. Mater. Interfaces.

[50] Osgood H., Devaguptapu S.V., Xu H., Cho J., Wu G. (2016). Transition metal (Fe, Co, Ni, and Mn) oxides for oxygen reduction and evolution bifunctional catalysts in alkaline media. Nano Today.

[51] Antoine O., Durand R. (2000). RRDE study of oxygen reduction on Pt nanoparticles inside Nafion: H_2_O_2_ production in PEMFC cathode conditions. J. Appl. Electrochem..

[52] Akram A., Freakley S.J., Reece C., Piccinini M., Shaw G., Edwards J.K., Desmedt F., Miquel P., Seuna E., Willock D.J. (2016). Gas phase stabilizer-free production of hydrogen peroxide using supported gold-palladium catalysts. Chem. Sci..

[53] Edwards J.K., Pritchard J., Lu L., Piccinini M., Shaw G., Carley A.F., Morgan D.J., Kiely C.J., Hutchings G.J. (2014). The direct synthesis of hydrogen peroxide using platinum-promoted gold–palladium catalysts. Angew. Chem. Int. Ed..

